# Non-destructive characterization of bone mineral content by machine learning-assisted electrochemical impedance spectroscopy

**DOI:** 10.3389/fbioe.2022.961108

**Published:** 2022-09-05

**Authors:** Aihik Banerjee, Youyi Tai, Nosang V. Myung, Jin Nam

**Affiliations:** ^1^ Department of Bioengineering, University of California, Riverside, University Ave, Riverside, CA, United States; ^2^ Department of Chemical and Biomolecular Engineering, University of Notre Dame, Notre Dame, IN, United States; ^3^ UC-KIMS Center for Innovative Materials, University of California, Riverside, University Ave, Riverside, CA, United States

**Keywords:** bone mineral content, electrochemical impedance spectroscopy, equivalent circuit model, machine learning, bone healing

## Abstract

Continuous quantitative monitoring of the change in mineral content during the bone healing process is crucial for efficient clinical treatment. Current radiography-based modalities, however, pose various technological, medical, and economical challenges such as low sensitivity, radiation exposure risk, and high cost/instrument accessibility. In this regard, an analytical approach utilizing electrochemical impedance spectroscopy (EIS) assisted by machine learning algorithms is developed to quantitatively characterize the physico-electrochemical properties of the bone, in response to the changes in the bone mineral contents. The system is designed and validated following the process of impedance data measurement, equivalent circuit model designing, machine learning algorithm optimization, and data training and testing. Overall, the systematic machine learning-based classification utilizing the combination of EIS measurements and electrical circuit modeling offers a means to accurately monitor the status of the bone healing process.

## Introduction

Critical-sized bone defects, generally characterized as a bone loss greater than two times the diameter of the specific bone, pose a significant clinical concern, requiring therapeutic interventions for proper healing (Nauth et al., 2018; Kobbe et al., 2020). Non-union bone healing, which often occurs during the treatment of critical-sized bone defects, is an especially challenging condition, requiring surgical intervention and frequently causing inefficient bone repair with suboptimal clinical outcomes (Wildemann et al., 2021). Thus, the management of critical-sized bone defects remains a major clinical orthopedic challenge and it requires novel and safe therapeutic strategies for enhanced bone regeneration.

Bone healing is a highly dynamic process and continuous monitoring of the efficacy of a therapeutic approach is crucial for ensuring optimal treatment. One promising strategy is to quantify the change in mineral content to correlate it with the healing status in the defect region. To date, however, effective real-time *in situ* monitoring systems for bone healing are highly primitive at best and non-existent at worst (Augat et al., 2014; Rani et al., 2020; Ernst et al., 2021). Current assessment modalities, including X-ray diagnostic radiography, photon absorptiometry, quantitative computed tomography, and magnetic resonance imaging, suffer from many limitations such as low sensitivity for bone mineral content, high cost, the requirement of trained personnel, standardization of image quality/quantification, and radiation overexposure risks (Hak et al., 2014; Wildemann et al., 2021). Moreover, these endpoint qualitative assessments are often subjective, and their accuracy is reliant on the clinician’s expertise (Morshed et al., 2008; Claes and Cunningham, 2009; Schwarzenberg et al., 2020).

In this regard, electrochemical impedance spectroscopy (EIS) provides a means to non-destructively assess the bone healing process by characterizing the electrical properties of the tissue. Several studies have demonstrated the feasibility of utilizing EIS for determining bone health, where tissue impedance changes at specific frequencies of the applied alternating current (AC) potential were well correlated to the radiographically determined status of bone regeneration (Kozhevnikov et al., 2016; Afsarimanesh et al., 2016; Dell’Osa et al., 2019a; Dell’Osa et al., 2019b). Particularly, Lin et al. utilized electrode implants to longitudinally monitor bone healing in murine fracture models, where the magnitude of impedance measurements was proportional to the quantified measures of bone volume and bone mineral density (Lin et al., 2015). Such tracking of the longitudinal changes of impedance with respect to those of non-fractured control samples allowed them to distinguish good healing as compared to non-union bone fracture (Lin et al., 2015). This *in vivo* application of EIS demonstrates the potential of the electrochemical analysis for bone fracture monitor and management in the clinic (Lin et al., 2019). However, this approach is semi-quantitative in nature, requiring separate control groups to assess the degree of bone healing.

Herein, we demonstrate the detection of bone mineral contents, a marker for the degree of bone healing, using the EIS technique empowered by machine learning models. Machine learning has emerged as an effective and accurate method to understand complex biological phenomena, especially human diseases and injuries (Yu et al., 2018; Liang et al., 2019; Tekkesin, 2019; Peng et al., 2021). Several studies have used various machine learning approaches to develop equivalent circuit models from EIS data (Tripathi and Maktedar, 2016; Cunha et al., 2019; Babaeiyazdi et al., 2021), but the application of machine learning in diagnosing the degree of bone health has not been attempted. The workflow presented in this study consisted of four main steps—an EIS impedance measurement, equivalent circuit modeling and data fitting, principal component analysis, and machine learning analysis—to gradually build up a bone composition detection strategy with the purpose of automatically formulating multiple impedimetric parameters into a recognition machine that determines the bone mineral content. Three different machine learning algorithms were compared in terms of their performance to categorize the mineral content of rat femur samples. Two types of datasets, one consisting of impedance data at different frequencies only and the other consisting of fitted equivalent circuit model parameters in addition to the impedance values, were used to evaluate the classification models in order to delineate the importance of the feature set used for multiclass classifications. We demonstrate that the machine learning-assisted EIS analysis enables the prediction of the bone mineral content with high accuracy, suggesting its potential for a real-time monitoring modality to assess the bone healing process.

## Methods

### Bone sample preparation

Femurs were excised from rat cadavers of similar size and age, surpluses from other non-skeletal studies. Bone samples of 6 mm in length, approximately twice the diameter of the as-extracted femurs, were prepared from the diaphysis of the femur by using a diamond saw and both ends were polished with sandpaper to ensure proper electrical contact between the electrode and the sample. For demineralization, the femur samples were incubated in 20% (v/v) Cal-Ex II decalcifier solution (Fisher Scientific) for varying durations to prepare samples containing a specific weight percentage of mineral. Specifically, bone samples having 0, 20, 40, 60, 80, and 100% of mineral contents (as compared to fully demineralized samples) were selected to simulate critical-sized bone defects at different healing stages. The wet weights of the bone samples were recorded after each treatment, which was used to calculate the weight percent of minerals remaining in the samples.

### Bone mineral content calculation

The bone mineral content, the wt% of mineral present in the samples after specific durations of demineralization, was indirectly calculated using the following formula,

$$W_{M,lost}=\rho_{M}\times V_{M,lost}=\;\rho_{M}\times \frac{\Delta W_{wet}}{\rho_{W}-\rho_{M}}$$

where $W_{M,lost}$ is the weight of mineral lost after decalcifier treatment, $\rho_{M}$ is the density of hydroxyapatite, which makes up the mineral phase of bone, $V_{M,lost}$ is the volume of mineral lost after decalcifier treatment, $\Delta W_{wet}$ is the change in the wet weight of the femur sample, and $\rho_{W}$ is the density of water. We assumed that the volume of mineral lost is replaced by the volume of media since buffer saturation was used to maintain the net volume.

### Surface characterization of bone samples

The morphology of the intact and demineralized bone samples was characterized using a VEGA3 scanning electron microscope (SEM) (Tescan Brno, Czech Republic). The bone samples were subject to a dehydration process for SEM sample preparation by their exposure to a graded ethanol series, followed by a graded ethanol-hexamethyldisilane series as previously described (Nam et al., 2007; Maldonado et al., 2016).

### Bone mineral content visualization

Alizarin red S staining (Sigma) was used to colorimetrically determine the mineral content in sectioned bone samples under various demineralization durations as previously described (Homer et al., 2019). The color intensity was quantified under each condition using ImageJ software and five slices were used for quantification for each condition.

### Electrochemical impedance spectroscopic (EIS) measurements

The electrochemical impedance measurements were carried out using a CH Instruments 604C electrochemical analyzer (CH Instruments Inc.). A custom-built sample holder and EIS measurement system, consisting of a mini-vise and gold-coated stainless steel disc electrodes with soldered insulated copper wires, was used for EIS signal acquisition. To maintain high humidity and avoid drying of the samples during measurement, a humidity chamber was used to encapsulate the entire measurement assembly. The bone sample was carefully positioned in between the two electrodes for uniform electrode-contact without applying excessive pressure on the sample during clamping. The EIS measurements were performed at 10 mV AC voltage to achieve a pseudo-linear system response (Habekost, 2021; Kretzschmar and Harnisch, 2021), and the impedance (Z) and phase angle (θ) were measured at sixty different frequencies in the range from 1 Hz to 100 kHz (10 data points per decade of frequency). Room temperature was maintained, and a humid chamber was used to prevent bone drying during the entire EIS measurement.

### Equivalent circuit modeling and data fitting

An equivalent circuit model was developed based on a physical interpretation of the electrochemical phenomena taking place in our electrochemical system. EIS Spectrum Analyzer software was used to fit the experimental data with the proposed equivalent circuit model. The Nelder-Mead algorithm was utilized for the fitting in order to determine the values of the equivalent circuit model components.

### Machine learning algorithms

Machine learning-based classification models were utilized for further analysis of data in order to set up a bone mineral content-based detection system. All the machine learning classifier models were established by the Python based open source visual programming software Orange toolkit (Bioinformatics Laboratory, University of Ljubljana). The detailed algorithm parameters are described in the Supplementary Material.

## Results

**FIGURE 1:**
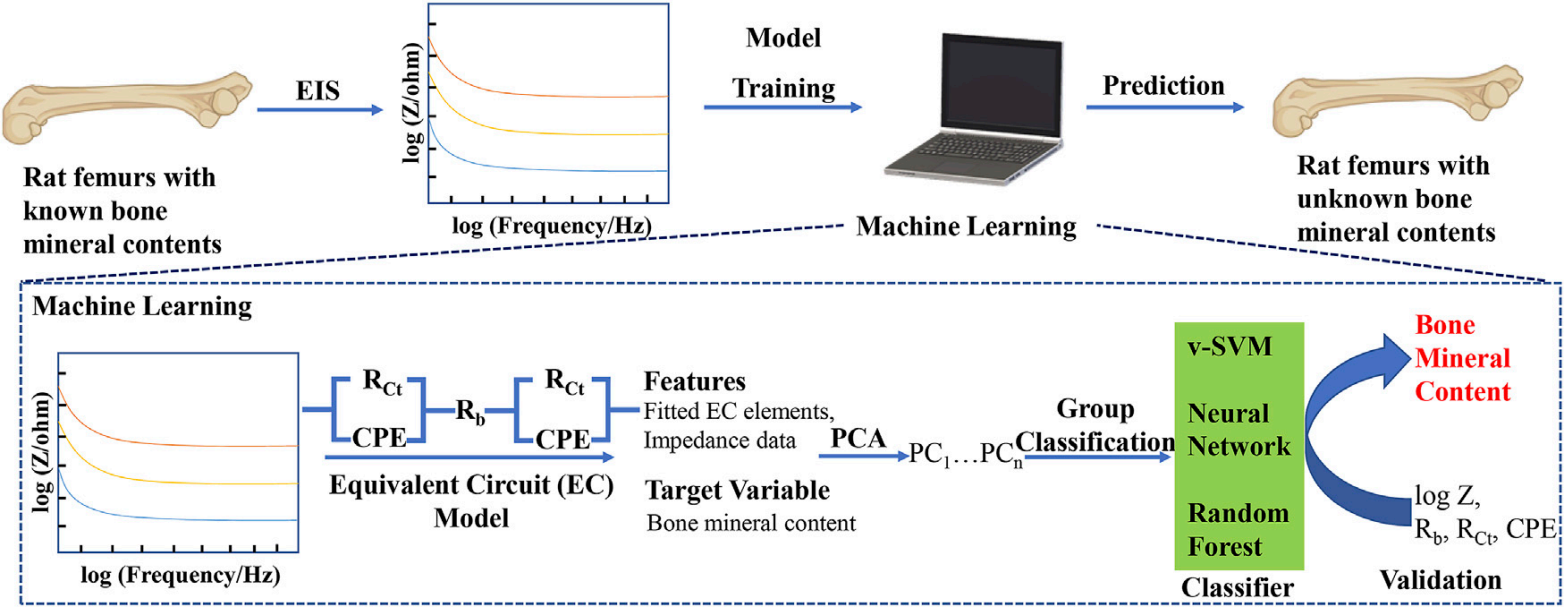
Schematic of machine learning-assisted electrochemical impedance spectroscopy (EIS) for the quantitative analysis of bone mineral content. PCA: Principal Component Analysis; v-SVM: Support Vector Machine.

The flowchart of our machine learning-assisted EIS strategy for the quantitative analysis of bone mineral content is shown in Figure 1. The key points in our approach include, 1) measurement of impedance data from the bone samples of defined mineral content, 2) using the measured impedance data to train and validate a machine learning model, 3) using the trained model to classify bone samples of unknown mineral composition. To the best of our knowledge, this approach of classifying bone samples using a combination of EIS and machine learning is the first used to analyze bone mineral contents, a marker for bone regeneration, potentially offering a non-destructive, quantitative method to track bone regeneration.

**FIGURE 2:**
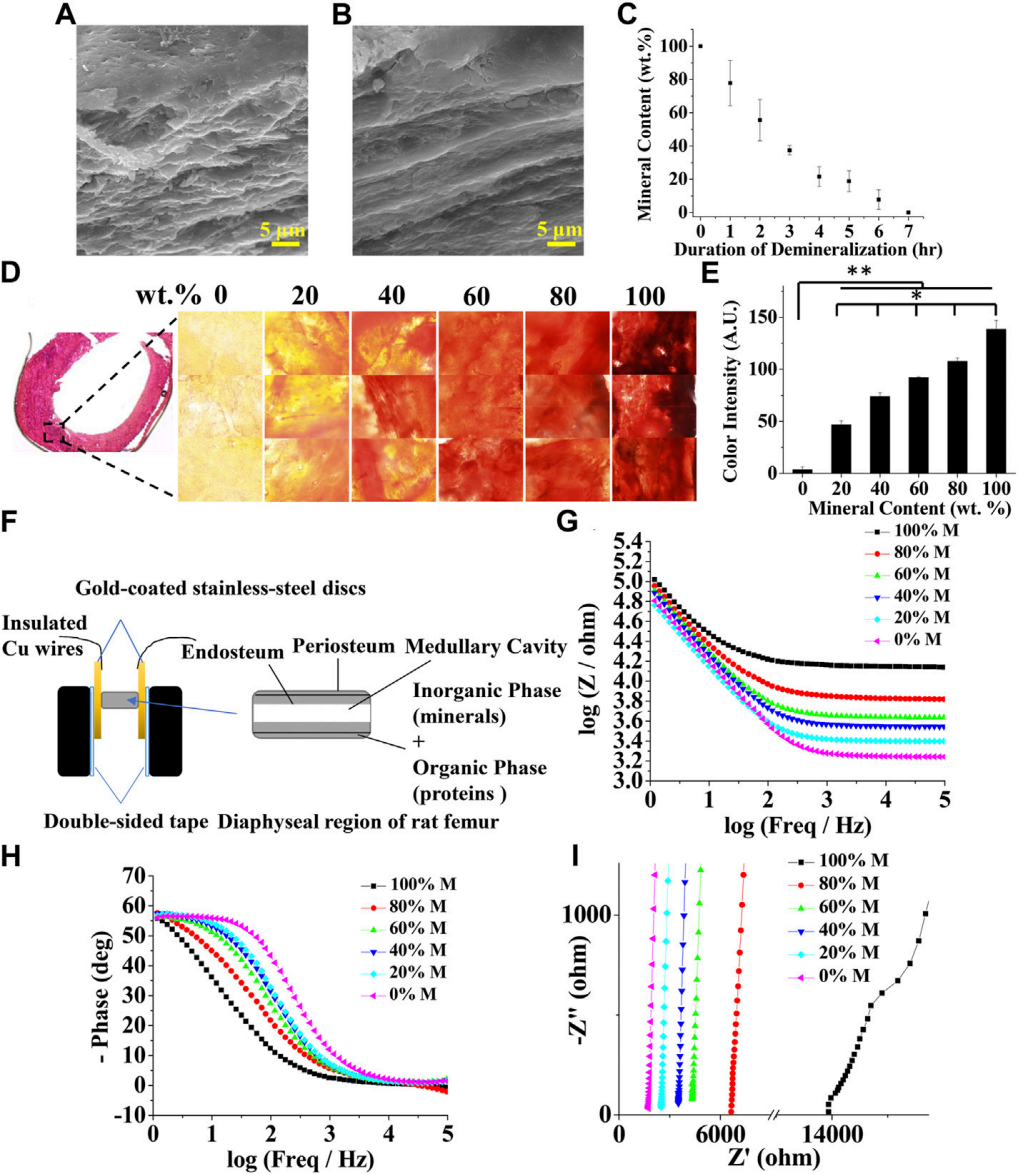
Characterization of bone mineral content and electrochemical impedance spectroscopy (EIS) at different levels of mineralization. Scanning electron microscopy (SEM) images of **(A)** intact **(B)** demineralized bone samples **(C)** Bone mineral content as a function of demineralization duration **(D)** Bone cryosection and alizarin red staining at 0, 20, 40, 60, 80, and 100 wt% bone mineral content **(E)** Quantified alizarin red staining intensity as a function of bone mineral content (*n* = 5, * and ** denote statistical significance of *p* < 0.05 and *p* < 0.01, respectively, analyzed by one-way ANOVA with Tukey’s posthoc test.) **(F)** Schematic showing an experimental setup for the measurement of EIS spectrum of a bone sample. Representative bone EIS spectra shown as **(G)** Bode magnitude **(H)** Bode phase angle, and **(I)** Nyquist plots.

To prepare samples of different mineral contents, rat femurs were treated with a demineralization solution for various durations. Fully decalcified bone samples showed a smooth fibrous structure owing to the remaining organic phase, mostly collagen in the bone as compared to intact bone samples (Figures 2A,B). The mineral content linearly decreased as the duration of the demineralization process increased, as shown in Figure 2C. This was further confirmed by alizarin red staining and its colorimetric quantification (Figures 2D,E). The impedances of bone samples with known mineral contents were measured in the longitudinal orientation as shown in Figure 2F. Representative Bode plots and Nyquist plots for bone samples with various mineral contents are shown in Figures 2G–I. As expected, EIS measurements showed a strong frequency dependency; the impedance was considerably higher at low frequencies than at high frequencies (Figure 2G). At lower frequencies, the signals are both resistive and capacitive (slopes of the curves in the Bode magnitude plot ≈45°, slanted lines), while at higher frequencies the signals become purely resistive with no capacitive contributions to the impedance (slopes of the curves ≈0°, parallel to the abscissa). This decrease is associated with a significant change in the phase shift. The phase is about 60° at low frequencies and drops to values close to zero when the frequency increases (Figure 2H). These observations are in agreement with the results of Balmer et al. (Balmer et al., 2018). The Nyquist plots also show that the real and imaginary components of the impedance decreased as the mineral content decreased, corroborating with the Bode plots (Figure 2I).

**FIGURE 3:**
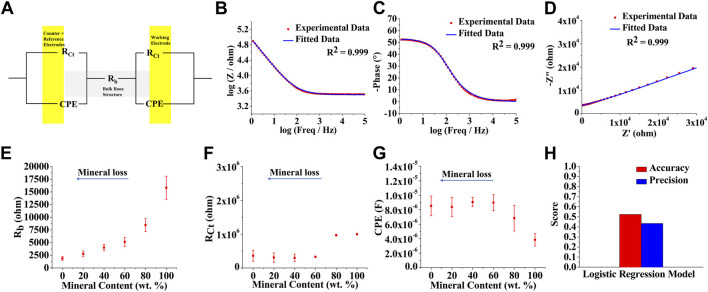
Equivalent circuit modeling for the factor deconvolution of electrochemical impedance spectroscopy (EIS) data **(A)** Equivalent circuit model used for fitting the bone EIS spectra. R_b_ = resistance from bulk bone structure, R_Ct_ = bone surface-metal electrode interfacial charge-transfer resistance, CPE = non-ideal double-layer capacitance (constant phase element) at the bone surface-metal electrode interface or contact region. Representative fitting results (20%) shown as **(B)** Bode magnitude **(C)** Bode phase angle, and **(D)** Nyquist plots. Equivalent circuit model-parametric characterization of bone samples at different levels of demineralization. Plots showing variation of **(E)** R_b_ **(F)** R_Ct_, and **(G)** CPE as a function of bone mineral content **(H)** Classification performance of a logistic regression discrimination model using impedance data and fitted equivalent circuit element data for training [*n* (total) = 70; *n* (0 wt%) = 12, *n* (20 wt%) = 12, *n* (40 wt%) = 12, *n* (60 wt%) = 12, *n* (80 wt%) = 9, *n* (100 wt%) = 13] and testing [*n* (total) = 21; *n* (0 wt%) = 4, *n* (20 wt%) = 4, *n* (40 wt%) = 4, *n* (60 wt%) = 3, *n* (80 wt%) = 3, *n* (100 wt%) = 3] datasets.

An equivalent circuit model was designed to describe the electrochemical processes of the EIS spectra and to deconvolute the impedance contributing factors by fitting the measured impedance data (Figure 3A). Based on the features of the EIS spectra, i.e., the presence of a single prominent peak in the Bode phase plots, a two-layer physical model was employed: bulk bone tissue, and bone surface-metal electrode interface. R_b_ represents resistance from bulk bone structure, while R_Ct_ and CPE represent interfacial charge-transfer resistance and non-ideal double-layer capacitance (constant phase element) at the bone surface-metal electrode interface, respectively (Figure 3A). The experimental data were fitted into the proposed equivalent circuit model and representative fitting results from the bone sample having a mineral content of 20%, including Bode magnitude, Bode phase angle, and Nyquist, are shown in Figures 3B–D, where the robust goodness of fit values, presented as *R* ^2^ values, has been achieved. A complete data sets for various mineral contents are shown in Supplementary Figures S1–S6.

In order to better understand and confirm the physical meanings of the proposed circuit model, the relationship between each equivalent circuit model parameter and corresponding mineral content was further investigated. As expected, the bulk bone structural resistance, R_b_, decreases with a decrease in bone mineral content (Figure 3E). Cortical bone mostly contains a mineral phase, which has large resistance, and hence demineralization results in a resistance drop. The interfacial resistance, R_Ct_, only drops after about 20% demineralization (or 80% mineral content) and then reaches a steady-state value, signifying that the interfacial resistance solely depends on the bone/electrode interfacial electrochemical effects and not on bone structural degradation (Figure 3F). In contrast, the CPE value increases slightly after about 20% mineral removal and then stabilizes, which is similar to the observation by Wang *et al.*, where the capacitance slightly decreased with increased apatite growth (Figure 3G) (Wang et al., 2003). The impedance values at different frequencies were combined with the fitted equivalent circuit parameters to test a simple regression model. Figure 3H shows the performance of the logistic regression model in predicting the bone mineral content, where the classification accuracy of 52.4% with a precision of 43.4% was observed.

**FIGURE 4:**
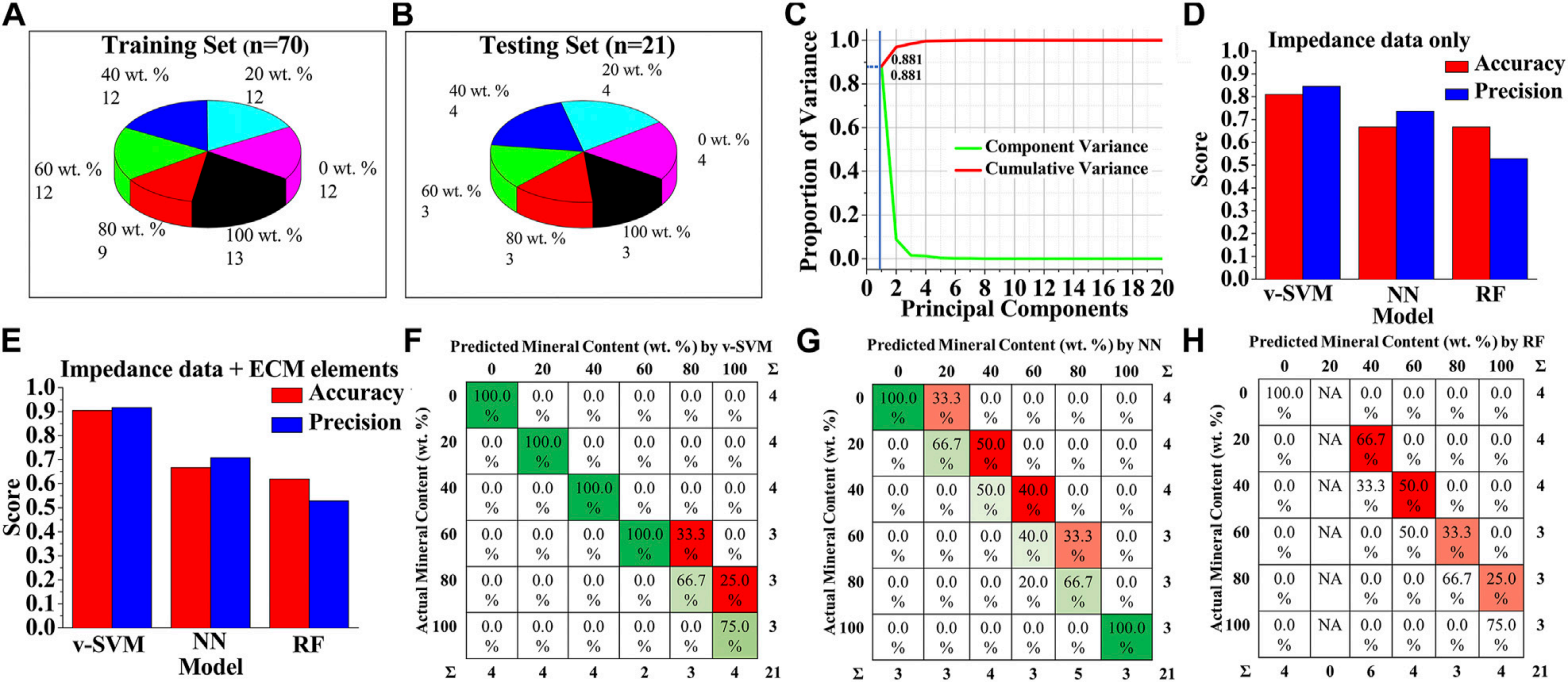
Machine Learning-based detection of bone mineral content **(A,B)** Data sets with different bone mineral contents, used for training and testing of machine learning models **(C)** Representative PCA scree plot showing the number of components used (blue line) along with proportion of variance explained. Classification performance of the three types of discrimination models when **(D)** only impedance data were used for training and testing, and **(E)** both impedance data and equivalent circuit-fitted element data were used for training and testing **(F–H)** Confusion matrix for **(F)** v-SVM **(G)** Neural Network, and **(H)** Random Forest algorithms for bone mineral content classification when both impedance data and equivalent circuit-fitted element data were used for training and testing.

To improve the prediction accuracy, various machine learning algorithms were employed. In order to establish an appropriate machine learning classification model and evaluate its performance, the original dataset was divided into a training set and a testing set, where the training set was used to establish prediction models and the testing set was used to verify the validity of the models (Figures 4A,B). A dataset of ninety-one measured impedance signals, corresponding to at least fifteen signals per mineral content category (0, 20, 40, 60, 80, 100 wt%), from multiple samples was prepared. Seventy data instances were randomly selected from the dataset, representing all the six classification categories, as the training dataset, and the remaining twenty-one measurements were utilized as the testing dataset. This dataset splitting ratio was chosen to ensure optimal classification performances of machine learning models (Xu and Goodacre, 2018; Thien and Yeo, 2021).

To avoid problems of overfitting, confusing the algorithms, and high computation cost, we first performed dimensionality reduction on the data using principal component analysis (PCA), instead of directly feeding the original data to the machine learning algorithms. As shown in Figure 4C, the first principal component contributed nearly 90% of the explained variance, and thus was chosen to represent the data with minimal loss of information. Scatter plot analysis of the principal components showed that the six classes of mineral content cannot be clearly distinguished due to the overlapping boundaries by PCA alone (Supplementary Figure S7). Hence, projections of all data features onto the first principal component dimension were then used as the input data for the machine learning models.

In order to find the best algorithm for the bone mineral content prediction, three different machine learning algorithm models—variant of support vector machine (v-SVM), neural network (NN), and random forest (RF) were trained based on the PCA-transformed dataset and compared, based on the classification accuracy and precision of correctly assigning categories to the instances in the testing. To further test the importance of the type of data used for model training, one dataset was prepared with only EIS impedance-frequency data, and another dataset contained a combination of fitted equivalent circuit model parameters and EIS impedance-frequency data. The predicting performances of v-SVM, NN, and RF classification algorithm models were compared, when only impedance values at 60 different frequencies in the range of 1 Hz–100 kHz were used as the features of the training and testing datasets (Figure 4D). Results showed that v-SVM exhibited the highest accuracy and precision scores as compared to the other algorithms; while the v-SVM predicted the mineral composition categories with 81% accuracy and 84.5% precision, the accuracy of prediction by the NN and the RF was 66.7% for both. The precision for NN and RF was 73.6 and 52.8%, respectively. Interestingly, when the values of the fitted equivalent circuit model parameters were used in addition to the impedance values as the feature set of training and testing datasets, the performance of the v-SVM markedly increased with classification accuracy reaching approximate 91% with a precision of about 92% (Figure 4E). In the case of the NN, the classification accuracy remained the same at 66.7% with a comparatively lower precision of 70.8%, while for the RF model, the accuracy dropped to 61.9% with the same precision of 52.8%. Therefore, among the three supervised algorithm models, v-SVM exhibited superior accuracy and precision to the other two methods as it can better identify the six classes of bone samples with different mineral levels, especially when the equivalent circuit modeling was employed. It should be also noted that there was a significant improvement in accuracy and precision by all these machine learning algorithms as compared to the logistic regression discrimination model (Figure 3H, Figures 4D,E). Figures 4F–H shows the confusion matrices for v-SVM, NN, and RF, respectively, where each row represents the actual mineral content of samples, and each column represents the predicted mineral content by the respective classification models. The detected values under the v-SVM method corresponded best to the raw data as compared to the other algorithm methods.

## Discussion

Treatments of the critical-sized bone defect are challenging due to the frequent surgical intervention and the high risk of causing non-union bone healing (Roddy et al., 2018; Stewart, 2019). To ensure optimal therapeutic treatment, monitoring the bone healing process is crucial. X-ray diagnostic radiography is one of the most used diagnosing and monitoring techniques in clinical settings (Wong et al., 2012). Limitations such as low accuracy, poor quantification, and radio safety, however, still exist. Quantitative computed tomography (QCT), on the other hand, provides a means to assess bone healing by providing high-resolution images and quantitatively measuring the bone mineral content (Augat et al., 1997). However, large signal noise, high cost, and limited accessibility have prevented its further application in the continuous monitoring of bone therapy.

In this regard, EIS provides a means to non-destructively assess bone healing process by characterizing the electrical properties of the tissue in relation to bone mineral content. We showed a decrease in the magnitude of impedance with the decrease in mineral content. In addition, the single peak in the Bode phase plots spreads over a wider frequency range with decreased mineral contents. The shift of this “peak”, corresponding to a time constant (R||C) of the system, indicates that the electrochemical process becomes faster during the progress of demineralization due to the removal of the resistive mineral phase. The observation of the raw impedance values of the bone to assess the mineral content, however, is still semi-quantitative, leading to inaccurate prediction of the mineral content from the overall impedance dataset.

Therefore, equivalent circuit modeling was employed to extract the individual contributing factors from the impedance, including the electrical components of bulk bone tissue and the bone-electrode interfaces. For the purpose of the equivalent circuit modeling, the cortical bones can be assumed to exhibit mostly a resistive behavior and that the bone/electrode phase boundaries result in the appearance of an interfacial capacitance, uncharacteristic of bulk bone tissue (Bauerle, 1969; Mercanzini et al., 2009; Jiang et al., 2016). Furthermore, since the cortical bone samples under study are mostly composed of an inorganic phase, which exhibits low relative permittivity <10, the capacitive behavior of the samples can be neglected for simplicity (Asgarifar, 2012). Apart from the capacitive behavior at the bone-electrode interface, which is typically seen at lower frequencies (<100 kHz), another capacitive contribution to the impedance spectra could result from the stray capacitance of the measurement system at higher frequencies (>100 kHz). However, since the range of frequencies used in this study was from 1 Hz to 100 kHz and precautions were taken to carefully insulate the measurement setup, stray capacitive contributions to the impedance spectra were neglected. In addition, bone tissues can be considered as an inhomogeneous composite material that contains a less conductive mineral phase (hydroxyapatite) and a more conductive hydrated organic phase (mostly collagen). Therefore, the justifications for using CPE instead of a capacitor are two-fold: 1) the inhomogeneity of bone composition coupled with contact-surface roughness, leading to pseudocapacitive behavior at the interface, and 2) a better fit of the simulated data with the experimental data. The conductive charge carriers in the electrochemical system under study are ions and electrons. Thus, the bone/electrode interfacial phenomena are represented by a parallel combination of R_Ct_ and CPE, which is then connected in series to the bulk bone structural resistance, R_b_. The interfacial charge transfer resistance R_Ct_ is the resistance for the electron to change the phase, *i.e.*, from the electrode into the hydrated tissue. The equivalent circuit model we designed successfully deconvolute the impedance contributing factors, yet the prediction accuracy solely based on these deconvoluted values remains low due to sample variability. This is especially true if outliers are present in the overall dataset, often observed in the clinical datasets. These experimental error-based subtle ambiguities in the overall dataset cannot be resolved by simple classifiers like regression models as shown in Figure 3H.

In this regard, we utilized the machine learning algorithm models, due to their automation and robustness, as an analytical solution for categorizing bone samples of different mineral contents with multiple impedimetric parameters. The impedance values and their deconvoluted factors obtained from the equivalent circuit model were processed using various algorithm models and the best prediction accuracy was achieved when using the v-SVM, as compared to the other algorithms, including neural network, and random forest. Although small errors still exist due to a relatively small sample size of data being used for training and testing, our approach of random extraction, training, and prediction of the testing data showed that the differences among the data obtained from each group had little effect on the overall results. These results thus indicate that an appropriate equivalent circuit model and an optimal machine learning approach are both necessary for the adaptability and accuracy in bone mineral content detection, providing a means to accurately monitor the healing process of bone.

In this brief research report, we have developed an analytical method combining EIS and machine learning for the quantitative assessment of bone mineral content. We demonstrate that the electrochemical parameters of the bone tissue correlated well with its composition. The classification ability of various algorithms using the EIS data was compared. The results show that the best comprehensive performance is obtained by SVM when equivalent circuit model data were incorporated into raw impedance data. By incorporating multiple impedimetric parameters, the machine learning model enables the accurate determination of bone mineral content. Due to the advantages in adaptability, automation, and accuracy, we anticipate that the method established in this study will find various applications in bone defect management. These results might help further progress on the rapid and longitudinal monitoring of bone healing status and could even be used for the detection and analysis of bone defects. Moreover, this work proves the application potential of machine learning tools in electrochemical research.

## Data Availability

The raw data supporting the conclusions of this article will be made available by the authors, without undue reservation.
