# Supplementary material for: Non-destructive characterization of bone mineral content by machine learning-assisted electrochemical impedance spectroscopy
Source: Front Bioeng Biotechnol. 2022 Sep 5;10:961108. doi: 10.3389/fbioe.2022.961108 (PMC9484274; doi:10.3389/fbioe.2022.961108)
Supplement: Supplementary file 1 [file DataSheet1.PDF]

Supplementary Material

Method

Machine learning algorithms:

The EIS dataset was randomly divided into 2 subsets – the training dataset of 71 instances, and the testing dataset of 21 instances. The raw dataset had 64 features – 60 different frequencies in the range from 1 Hz to 100 kHz and 4 equivalent circuit model fitted parameters. In order to reduce the dimensionality of the feature set with minimal loss of information, principal component analysis (PCA) was first employed prior to feeding the training data into various supervised classification models, namely Support Vector Machine (SVM), Neural Network (NN), and Random Forest (RF). The first principal component, explaining about 90% of the variance while avoiding fitting issues, was used as input for the different classification models, and the bone mineral content was used as the model target variable or classification output. Each model was optimized towards the best prediction performance by iteratively trying every probable combination of classifier function parameters and penalty factors. Briefly, the radial basis function (RBF) was chosen as the kernel function for the ν-SVM algorithm due to its great advantage in solving multiclass classification problem (Zhang et al., 2018, Zhu et al., 2019). To obtain an optimal ν-SVM model, the regression cost C and complexity bound ν for the RBF function were set at 1.00 and 0.20, respectively, optimized by testing different combinations and the best C and ν were determined by the accuracy of cross-validation. A numerical tolerance of 0.0016, which resulted in the best classification performance according to our preliminary study, was used with no iteration limit. For setting up an optimally performing artificial NN model, 5 hidden layers comprising 100 neurons each were used with rectified linear unit (ReLU) activation function and L-BFGS-B solver. The regularization parameter, α, was set at 0.02 and the maximal number of iterations was set at 250 with replicable training. The RF classifier was optimal when the number of trees was set at 10 with replicable training and subsets smaller than 2 were not split. These parameters were determined from systematic and rigorous optimization by surveying all the available solver/activation/kernel functions/relevant parameters for the respective models to yield the best classification performances without compromising calculation speed.

Supplementary Figures

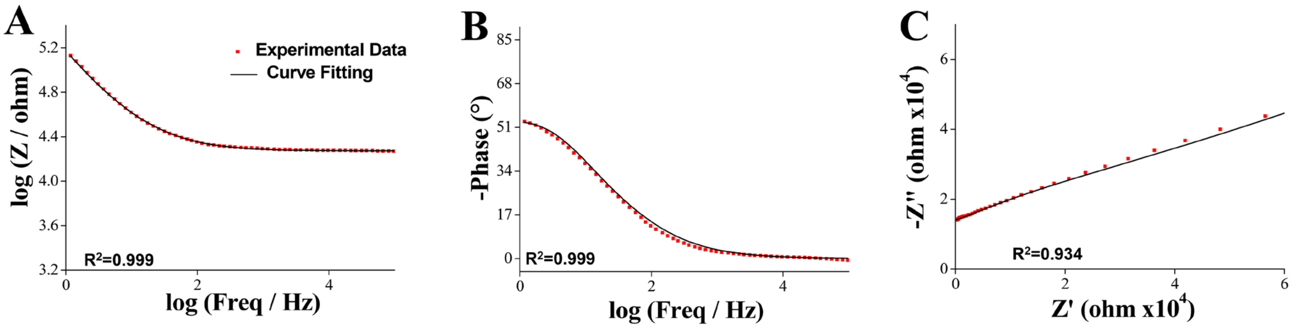

Figure S1. Representative fitting results of bone samples having 100% mineral content shown as (A) Bode magnitude, (B) Bode phase angle, and (C) Nyquist plots.

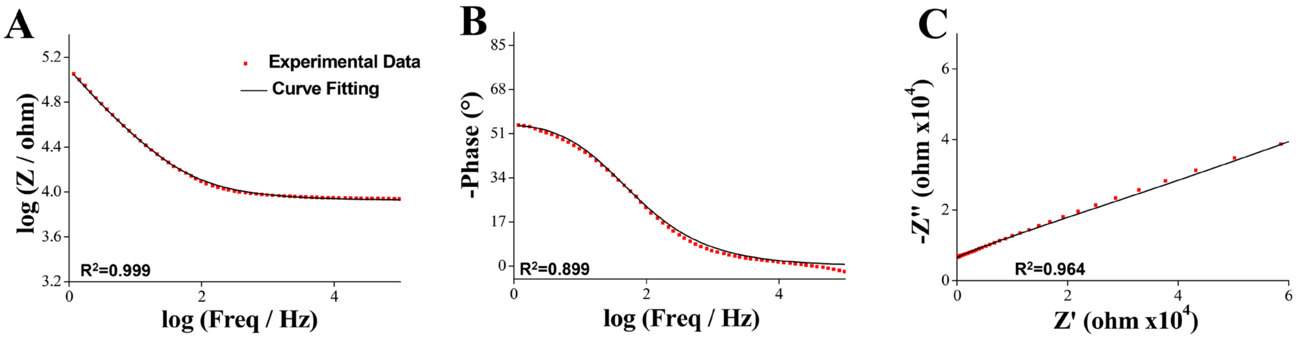

Figure S2. Representative fitting results of bone samples having 80% mineral content shown as (A) Bode magnitude, (B) Bode phase angle, and (C) Nyquist plots.

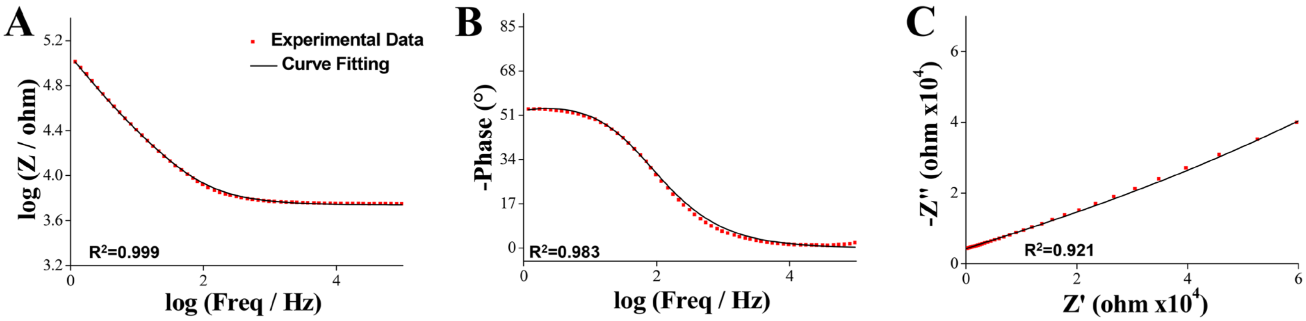

Figure S3. Representative fitting results of bone samples having 60% mineral content shown as (A) Bode magnitude, (B) Bode phase angle, and (C) Nyquist plots.

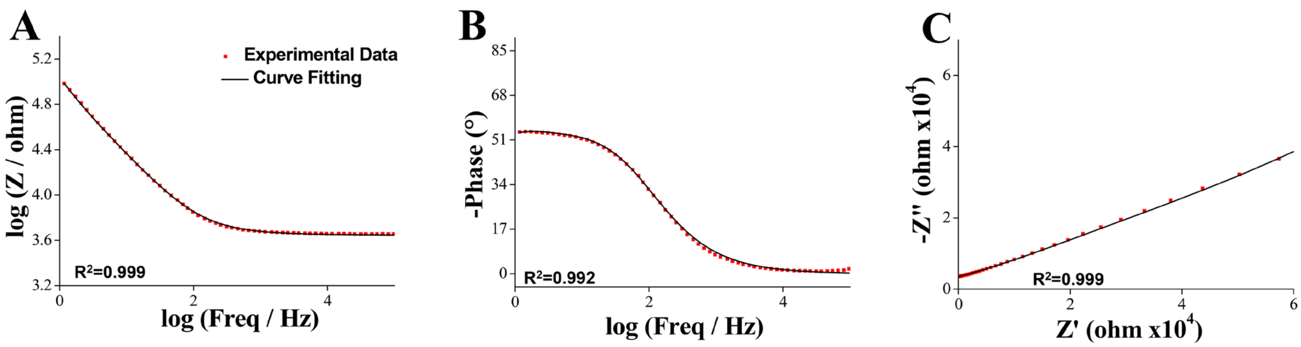

Figure S4. Representative fitting results of bone samples having 40% mineral content shown as (A) Bode magnitude, (B) Bode phase angle, and (C) Nyquist plots.

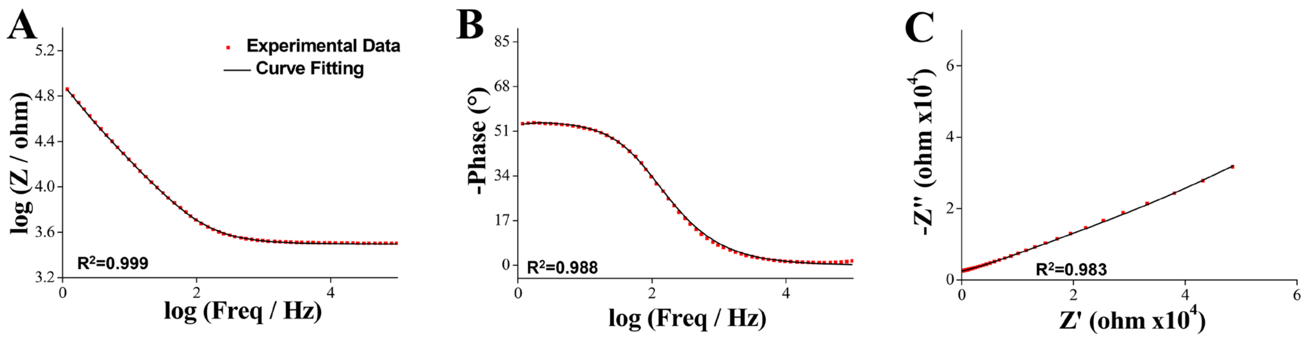

Figure S5. Representative fitting results of bone samples having 20% mineral content shown as (A) Bode magnitude, (B) Bode phase angle, and (C) Nyquist plots.

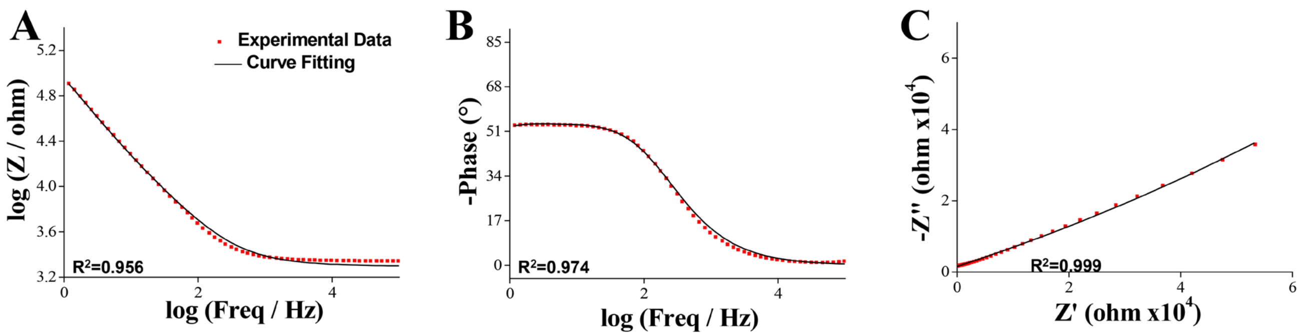

Figure S6. Representative fitting results of bone samples having 0% mineral content shown as (A) Bode magnitude, (B) Bode phase angle, and (C) Nyquist plots.

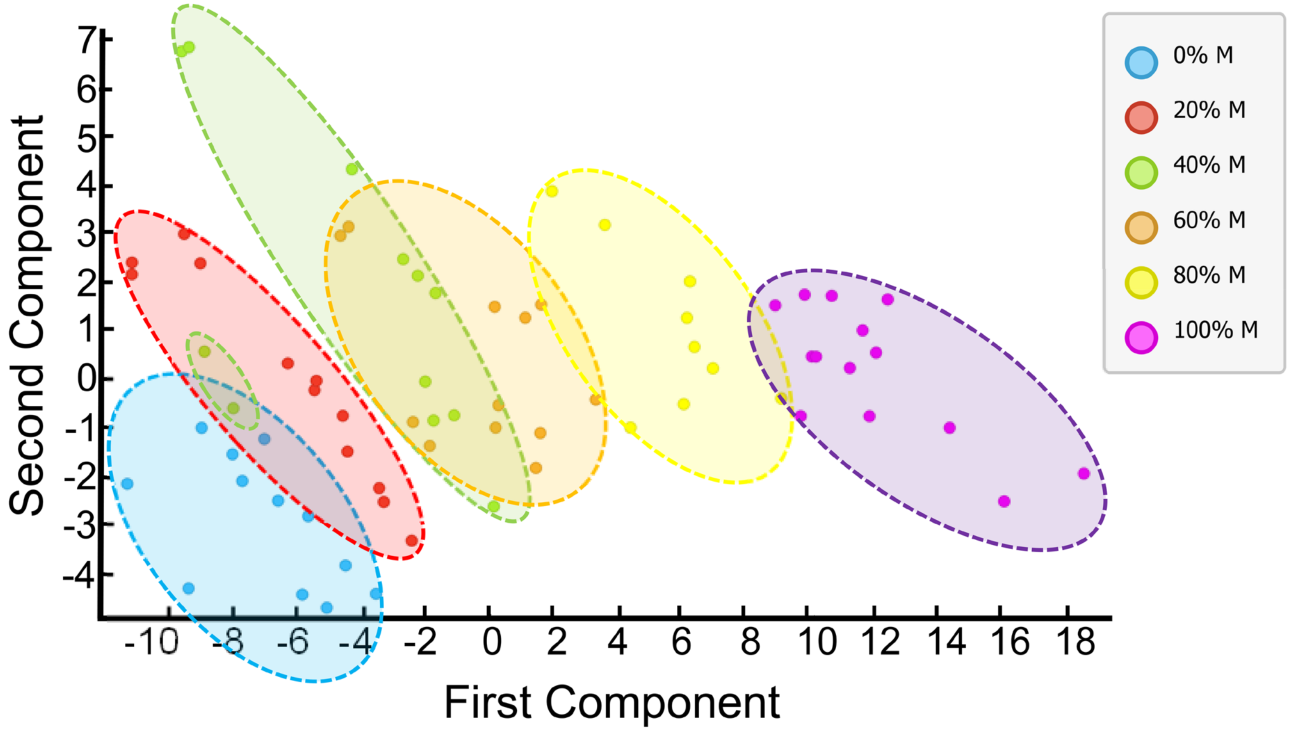

Figure S7. Scatter plot using principal component analysis (PCA) showing 6 clusters where the regions and boundaries were indicated by dashed line ellipses. M: mineral content.

References:

ZHANG, H., SUN, H., WANG, L., WANG, S., ZHANG, W. & HU, J. 2018. Near infrared spectroscopy based on supervised pattern recognition methods for rapid identification of adulterated edible gelatin. *Journal of Spectroscopy,* 2018.

ZHU, S., SUN, X., GAO, X., WANG, J., ZHAO, N. & SHA, J. 2019. Equivalent circuit model recognition of electrochemical impedance spectroscopy via machine learning. *Journal of Electroanalytical Chemistry,* 855**,** 113627.
